# Triglyceride deposit cardiomyovasculopathy as a predictor of vascular failure after coronary intervention in patients with hemodialysis: a preliminary analysis

**DOI:** 10.1080/0886022X.2026.2650580

**Published:** 2026-04-22

**Authors:** Masanobu Fujimoto, Yusuke Nakano, Ken-ichi Hirano, Tomohiro Onishi, Hirohiko Ando, Hirofumi Ohashi, Akinori Satake, Katsuhisa Waseda, Hiroshi Takahashi, Tetsuya Amano

**Affiliations:** ^a^ Department of Cardiology, Aichi Medical University, Nagakute, Japan; ^b^ Department of Triglyceride Science, Graduate School of Medicine, The University of Osaka, Suita, Japan; ^c^ Department of Cardiology, Narita Memorial Hospital, Toyohashi, Japan; ^d^ Cardiology, Fujita Health University School of Medicine, Toyoake, Japan

**Keywords:** Hemodialysis, percutaneous coronary intervention, drug-eluting stent, late loss, restenosis, triglyceride deposit cardiomyovasculopathy

## Abstract

Patients on hemodialysis (HD) are at high risk of vascular failure after percutaneous coronary intervention (PCI), even with new-generation drug-eluting stents (DES). Triglyceride deposit cardiomyovasculopathy (TGCV), a recently recognized cardiac metabolic disorder, features defective intracellular triglyceride hydrolysis with diffuse, concentric coronary narrowing, which may attenuate DES efficacy through distinct vascular remodeling biology, but its clinical impact remains unclear. This is a subanalysis of a retrospective, single-center study evaluating the prevalence and clinical outcomes of TGCV among HD patients. We included HD and catheter database data between April 2011 and March 2017. Among 118 patients suspected coronary artery disease, 26 patients who underwent [123I]-β-methyl-iodophenyl-pentadecanoic acid scintigraphy and a 12-month follow-up coronary angiography after DES implantation were analyzed. Patients were divided into the TGCV or non-TGCV group. The primary endpoint was in-stent late loss. The secondary endpoints were binary in-stent restenosis (ISR) and target lesion revascularization (TLR). Nine patients (34.6%) had TGCV. In-stent late loss was higher in the TGCV group than in the non-TGCV group (median 1.17 mm versus 0.29 mm; *p* < 0.001), with significantly higher ISR and TLR (58.3% versus 9.5%; *p* = 0.005; adjusted odds ratio [OR] 18.1, 95% confidence interval [CI] 2.23–146 and 50.0% versus 9.5%; *p* = 0.015; adjusted OR 12.3, 95% CI 1.62–93.2, respectively). TGCV was associated with higher vascular failure after DES implantation in HD patients, and might represent a potential therapeutic target after PCI. Given the small cohort and wide CIs, these findings are preliminary and hypothesis-generating, warranting validation in larger, prospective studies.

## Introduction

Despite the development of medical therapies and therapeutic procedures, such as statins and percutaneous coronary intervention (PCI), patients on hemodialysis (HD) are at a higher risk of cardiovascular events [1]. Given the poor response to any invasive revascularization and medical therapies, vascular failure after PCI, including in-stent late loss and target lesion revascularization (TLR), remains high among dialysis patients even for the new-generation drug-eluting stent (DES) era [2–5].

Triglyceride deposit cardiomyovasculopathy (TGCV) is a cardiovascular disorder, first reported in Japanese patients [6]. In TGCV, the defective hydrolysis of intracellular triglyceride (TG) leads to a novel form of diffuse, narrowing atherosclerosis causing TG deposition in the smooth muscle and endothelial cells in advanced cases, as against atherosclerosis induced by hypercholesterolemia presenting with focal and eccentric stenosis and cholesterol-laden macrophages [6–9]. Recently, an overview of TGCV has been incorporated into the Japanese heart failure guidelines, reflecting growing clinical recognition of this condition [10].

We recently reported that TGCV may be latent in HD patients and associated with poor clinical outcomes [11]. TGCV—a disorder of defective intracellular TG hydrolysis leading to diffuse, concentric coronary narrowing—may represent a distinct substrate for DES failure. However, whether TGCV confers excess late loss and clinically relevant vascular failure after DES implantation in HD patients remains unknown. Given these pathological characteristics and poor response to contemporary therapies, we hypothesized that coexisting TGCV in HD patients would contribute to adverse outcomes after PCI [11–13]. Therefore, we conducted the first preliminary study to evaluate the impact of TGCV on vascular failure after new-generation DES implantation in patients on HD.

## Methods

### Study design and population

**Figure 1.**
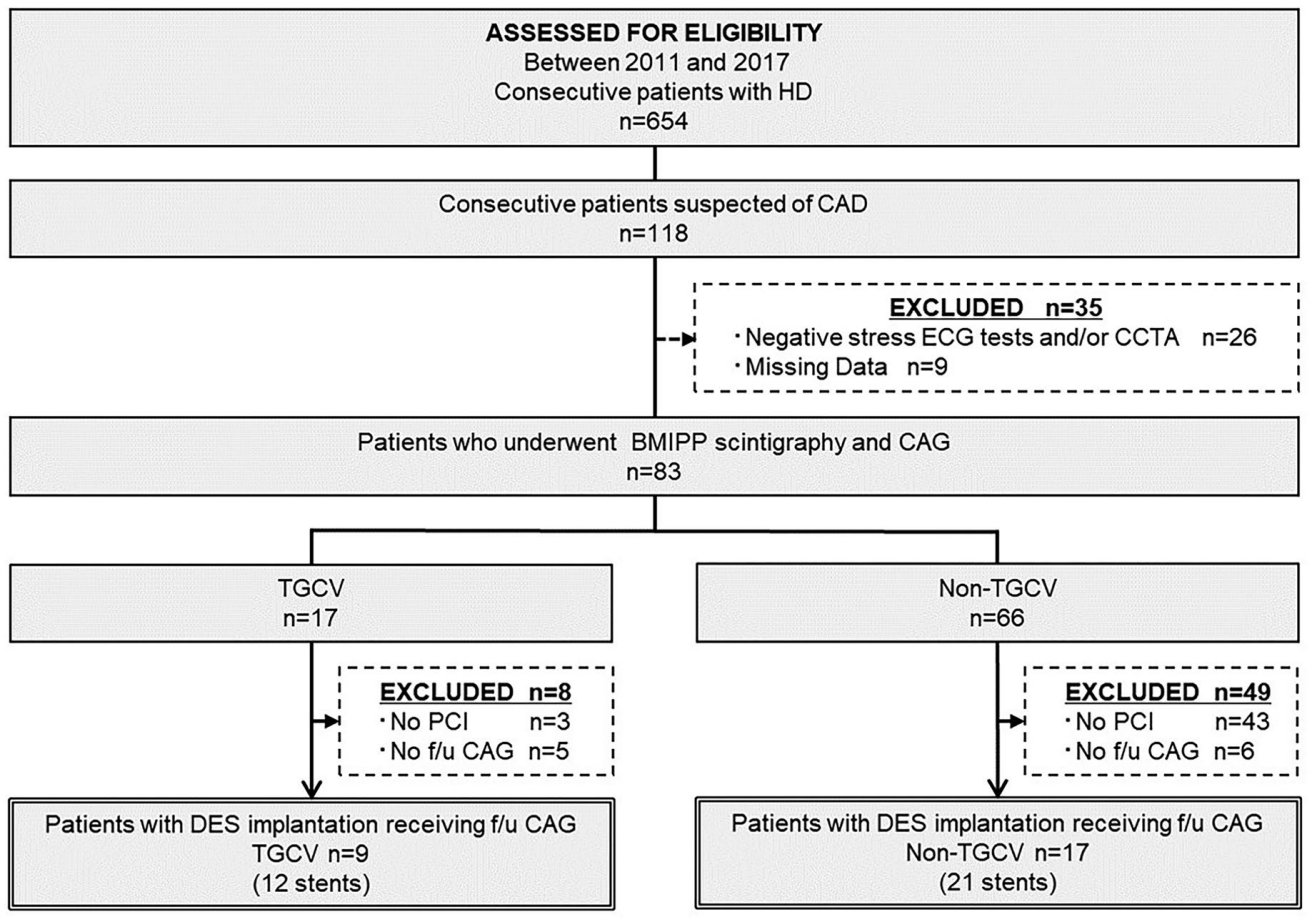
Patient flow chart. In total, 654 consecutive patients with HD between April 2011 and March 2017 were retrospectively assessed for eligibility. Of which, 118 cases were suspected of CAD; 26 cases with no abnormalities on exercise ECG and/or coronary CT and 9 cases with missing data were excluded. Consequently, 83 patients who underwent BMIPP scintigraphy and CAG to diagnose CAD were selected because these data are simultaneously required to diagnose TGCV based on the latest diagnostic criteria for TGCV published in 2020 by the Japan TGCV study group. Finally, data from 26 patients with DES implantation who received follow-up CAG were extracted and allocated accordingly, with the TGCV group consisting of 9 patients retrospectively diagnosed with TGCV and the non-TGCV group consisting of 17 non-TGCV patients. Abbreviations: BMIPP, [123I]-β-methyl-iodophenyl-pentadecanoic acid; CAD, coronary artery disease; CAG, coronary angiography; CCTA, coronary computed tomography angiography; DES, drug-eluting stent; f/u CAG, follow-up coronary angiography; HD, hemodialysis; PCI, percutaneous coronary intervention; TGCV, triglyceride deposit cardiomyovasculopathy.

The present study was a sub-analysis of a previous cohort study that evaluated the prevalence and clinical outcomes of TGCV among patients on HD [11]. The retrospective observational study included data from the cardiac catheter database of Narita Memorial Hospital between April 2011 and March 2017 [11]. Figure 1 describes the process of patient selection. Briefly, among 654 consecutive patients on HD during the follow-up period, 118 patients were suspected of having coronary artery disease (CAD) based on clinical findings. Of those, data from 83 patients who underwent [123I]-β-methyl-iodophenyl-pentadecanoic acid (BMIPP) scintigraphy and coronary angiography (CAG) were analyzed. BMIPP scintigraphy and CAG, regardless of the BMIPP scintigraphy results, were performed as part of the routine protocol to diagnose CAD [11]. This section outlines the patient selection flow of the original study from which the present sub-analysis was derived.

In the present cohort study, to assess 1-year PCI outcomes—including in-stent late loss, binary in-stent restenosis (ISR), and TLR—data from patients with DES implantation who underwent follow-up CAG were analyzed and patients were divided into the TGCV or non-TGCV group. TGCV was diagnosed based on the diagnostic criteria presented in 2020 (Supplemental Table S1) [10,14].

Inclusion criteria for the present analysis were:

(1) maintenance HD, (2) suspected CAD undergoing both BMIPP scintigraphy and diagnostic CAG per institutional protocol, (3) PCI with implantation of at least one new-generation DES, and (4) planned protocolized follow-up CAG at 12 months.

Exclusion criteria were: (a) no PCI performed, (b) PCI without DES, (c) lack of follow-up CAG, or (d) missing core QCA data. Of 83 patients with both BMIPP and CAG, 26 met all criteria.

The present study complied with the following diagnostic items of the criteria:

- Essential items for assessing impaired long-chain fatty acids metabolism or TG deposition in the myocardium: decreased washout rate (WOR) of BMIPP of <10% in [123I]-BMIPP scintigraphy, as well as some other items.
- Major items: decreased left ventricular ejection fraction (<40%), diffuse narrowing of the coronary arteries documented by CAG and/or coronary CT angiography, and other items.

BMIPP scintigraphy is the only practical method for the diagnosis of TGCV because the WOR of BMIPP reflects myocardial TG metabolism (Supplemental Text S1). Diffuse narrowing of the coronary arteries can be defined as ‘consecutive or longitudinal’ and ‘complete or partial’ obstruction in coronary vessels [11]. To validate the coronary obstruction burden on CAG, we compared 30 randomly selected coronary angiograms [11]. Two interventional specialists (H.A. and Y.N.) independently interpreted the coronary angiograms. TGCV was confirmed when one or more essential items and one or more major items were met.

### Ethics statements

This cohort study was conducted in accordance with the Declaration of Helsinki. The study protocol was approved by the Ethics Committee of Narita Memorial Hospital (approval number: R1-23-02), and written informed consent was obtained from all patients or their family members.

### Assessment of clinical outcomes

The primary endpoint was in-stent late loss up to 12 months after PCI; this was defined as the difference between the minimal lumen diameter (MLD) immediately after PCI and the MLD on follow-up CAG. The secondary endpoints were ISR and TLR. ISR was defined as angiographic luminal diameter narrowing ≥50% anywhere within the stent and/or within the 5-mm borders proximal or distal to the stent, as assessed using quantitative coronary angiography (QCA). Follow-up CAG was usually performed 12 months after PCI, even in patients without ischemic symptoms; time-to-event data were not systematically collected, and survival analyses were therefore not performed. TLR was defined as revascularization of the target lesion, either after PCI or after coronary artery bypass grafting, diagnosed based on clinical and physiological assessments. TLR was considered if re-intervention of the target lesion was required due to symptomatic stenosis ≥50% of the diameter during follow-up and ischemic change on electrocardiography and fractional flow reserve assessment, with an inducible ischemia cutoff value of 0.75. Coronary lesion types were based on the American Heart Association/American College of Cardiology classification. Cardiovascular events, including myocardial infarction, heart failure, cerebral hemorrhage, and sudden death during the follow-up period were also assessed.

### Quantitative coronary angiography analysis

Contrast-filled guide catheters were used as reference standards in the QCA analysis. Matched end-diastolic frames of the angiograms before and after PCI and at follow-up were analyzed by contour detection using the minimum cost algorithm QCA-Cardiovascular Measurement System (version 3.0, MEDIS, Leiden, The Netherlands). Cine frames for catheter calibration or vessel QCA analysis were selected by an experienced QCA analyst (H.A.). All vessel measurements for the selected frames were performed by 2 experienced QCA analysts with similar laboratory experience (H.A. and Y.N.).

### Statistical analysis

Continuous variables with a normal distribution are expressed as mean ± standard deviation, and inter-group comparison was performed using the unpaired Student’s t-test. Normality of the variables was assessed, and those failing the normality tests were expressed as medians with interquartile ranges and compared using the Mann–Whitney U test. Categorical variables are presented as patient number (%) and analyzed using Fisher’s exact test. The Cohen kappa coefficient was used to measure inter-rater reliability for diffuse narrowing of the coronary arteries. The in-stent late loss was compared between the two groups using the Fisher exact test and analysis of covariance (ANCOVA). Multivariate logistic regression analysis was performed to assess the predictors of ISR and TLR adjusted for confounding factors (age and sex) in model 1. Various factors known as classical risk factors of ISR and TLR were adjusted for models 2, 3, and 4. Given low event counts, multivariable logistic models were specified parsimoniously and interpreted as exploratory; primary inference relies on univariable estimates with attention to effect direction and uncertainty. Because this retrospective subanalysis used a fixed historical cohort, no *a priori* sample size calculation was performed; the study is exploratory. Statistical significance was set at *p* < 0.05. All statistical analyses were performed using IBM SPSS Statistics for Windows, version 25 (IBM Corp., Armonk, NY, USA).

## Results

### Baseline patient characteristics

The study cohort included 9 and 17 patients in the TGCV and non-TGCV groups, respectively. The baseline characteristics of the patients are shown in Table 1. There were no significant inter-group differences in terms of patient characteristics, except for the prevalence of sex, and the WOR of BMIPP associated with the TGCV diagnostic criteria. Cohen’s kappa coefficient for the diffuse narrowing of the coronary arteries was 0.86.

**Table 1.** Patient characteristics.

Variable	TGCV	Control	p-value
No. of patients, n	9	17	
Male sex, n (%)	5 (55.6)	16 (94.1)	0.035
Age, years	69.7 ± 9.0	63.4 ± 9.6	0.119
BMI, kg/m^2^	21.3 ± 5.2	23.6 ± 5.5	0.320
Prior MI, n (%)	1 (11.1)	3 (17.6)	>0.999
Prior CABG, n (%)	1 (11.1)	0 (0.0)	0.346
Hemodialysis periods, years	6 (5, 8)	2 (2,6)	0.332
Hypertension, n (%)	6 (66.7)	15 (88.2)	0.302
Diabetes Mellitus, n (%)	4 (44.4)	12 (70.6)	0.234
Dyslipidaemia, n (%)	6 (66.7)	9 (52.9)	0.683
Current smoker, n (%)	5 (55.6)	8 (47.1)	>0.999
Statin use, n (%)	5 (55.6)	9 (52.9)	>0.999
Insulin use, n (%)	2 (22.2)	5 (29.4)	>0.999
DAPT use, n (%)	9 (100.0)	17 (100.0)	>0.999
P2Y12 inhibitor use, n (%)	7 (72.8)	17 (100.0)	0.111
BMIPP wash-out rate, %	2.3 (−7.6, 8.8)	17.2 (12.8, 24.9)	<0.001
BMIPP wash-out rate <10%, %	9 (100.0)	2 (11.8)	<0.001
Diffuse narrowing coronary arteries, n (%)	9 (100.0)	12 (70.6)	0.129
LVEF, %	64.3 ± 12.6	60.3 ± 11.6	0.798
LVEF < 40%, n (%)	1 (11.1)	0 (0.0)	0.346

Abbreviations: BMI, body mass index; BMIPP, [123I]-β-methyl-iodophenyl-pentadecanoic acid; CABG, coronary artery bypass grafting; DAPT, dual antiplatelet therapy; LVEF, left ventricular ejection fraction; MI, myocardial infarction; no., number; TGCV, triglyceride deposit cardiomyovasculopathy.

### Baseline lesion characteristics and quantitative coronary angiography analysis

In total, 33 lesions (TGCV group, *n* = 12; non-TGCV group, *n* = 21) treated using a DES were assessed (Table 2). Lesions per patient were 1 (IQR 1–2) in the TGCV group and 1 (IQR 1–1.5) in the non-TGCV group. Median periods of CAG follow-up were similar in both groups (11.4 months vs. 12.2 months; *p* = 0.389). Although there were no significant inter-group differences in terms of MLD at baseline, those at follow-up were significantly smaller in the TGCV group than in the non-TGCV group (1.55 ± 0.99 mm vs. 2.31 ± 0.80 mm; *p* = 0.023). Accordingly, percent diameter stenosis was significantly higher in the TGCV group than in the non-TGCV group (46.7 ± 29.3% vs. 23.2 ± 21.0%; *p* = 0.012).

**Table 2.** Lesion characteristics and QCA.

Variable	TGCV	Control	p-value
Lesion characteristic			
No. of lesions, n	12	21	
No. of lesions per patient	1 (1, 2)	1 (1, 1.5)	0.624
Target vessel, n (%)			
RCA	5 (41.7)	4 (19.0)	0.312
LAD	4 (33.3)	12 (57.1)	
LCX	3 (25.0)	5 (23.8)	
AHA/ACC Type B2/C, n (%)	12 (100.0)	18 (85.7)	0.284
Bifurcation lesion, n (%)	3 (25.0)	11 (52.4)	0.160
Type of DES, n (%)			
Sirolimus-eluting stent	1 (8.3)	0 (0.0)	0.276
Paclitaxel-eluting stent	0 (0.0)	1 (4.8)	
Zotarolimus-eluting stent	1 (8.3)	4 (19.0)	
Everolimus-eluting stent	10 (83.3)	13 (61.9)	
Biolimus-eluting stent	0 (0.0)	3 (14.3)	
Stent length, mm	18.9 ± 6.2	19.4 ± 5.8	0.830
Stent diameter, mm	3.00 ± 0.35	3.13 ± 0.40	0.354
QCA data			
Lesion length, mm	14.2 ± 4.5	14.4 ± 5.2	0.913
Reference diameter, mm	2.98 ± 0.54	3.09 ± 0.50	0.578
Pre MLD, mm	0.74 ± 0.32	1.04 ± 0.48	0.071
Pre %DS, %	70.5 ± 12.0	63.2 ± 16.6	0.193
Post MLD, mm	2.75 ± 0.52	2.81 ± 0.58	0.776
Post %DS, %	11.9 ± 5.3	11.6 ± 6.9	0.906
F/U MLD, mm	1.55 ± 0.99	2.31 ± 0.80	0.023
F/U %DS, %	46.7 ± 29.3	23.2 ± 21.0	0.012
Duration to F/U CAG, months	11.4 (8.7, 12.7)	12.2 (12.2, 12.2)	0.389

Abbreviations: ACC, American College of Cardiology; AHA, American Heart Association; CAG, coronary angiography; DES, drug-eluting stent; DS, diameter stenosis; F/U, follow-up; LAD, left anterior descending artery; LCX, left circumflex artery; MLD, minimal lumen diameter; Post, postoperative; Pre, preoperative; QCA, quantitative coronary angiography; RCA, right coronary artery; TGCV, triglyceride deposit cardiomyovasculopathy.

### In-stent late loss

**Figure 2.**
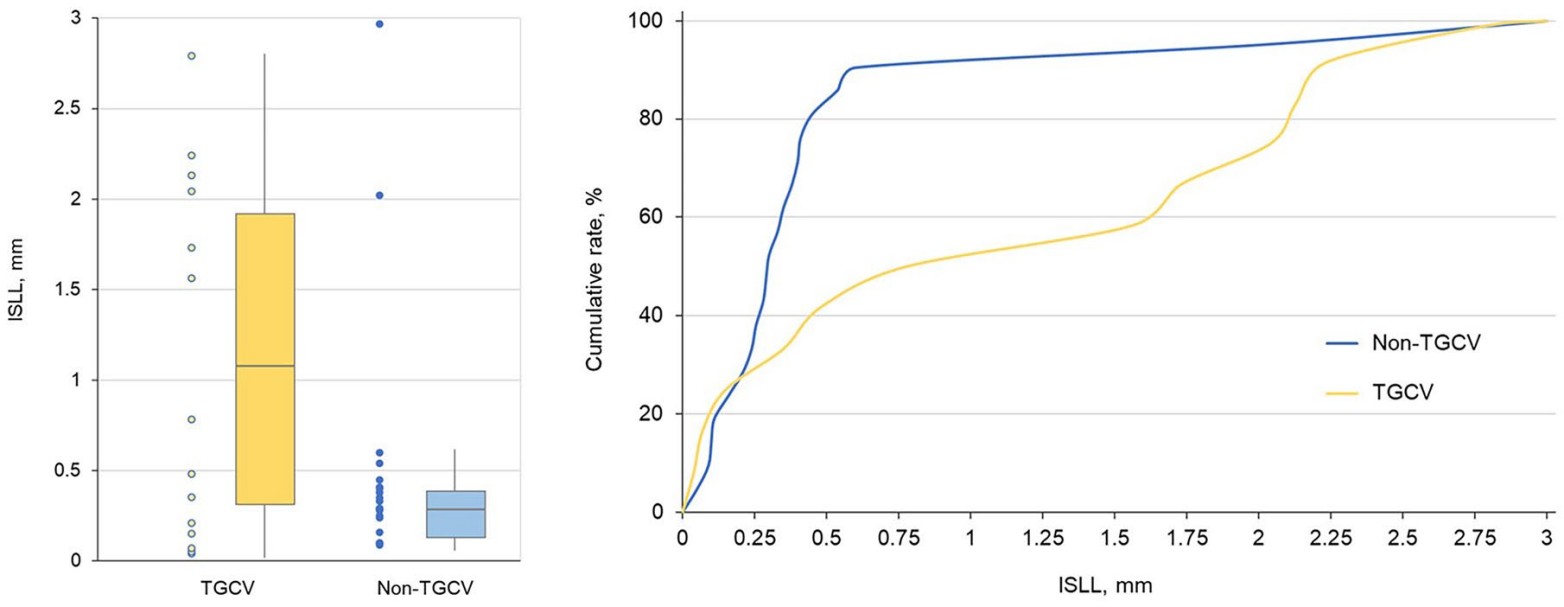
ISLL between the TGCV and non-TGCV groups. (a) The box plot indicates that ISLL was significantly higher in the TGCV group than in the non-TGCV group (1.17 mm [IQR: 0.30, 2.06] versus 0.29 mm [IQR: 0.21, 0.41], respectively; P = 0.045). The P-value was calculated using ANCOVA adjusted for sex and TGCV diagnosis. (b) The cumulative distribution curves of ISLL indicate a greater distribution of ISLL in the TGCV group than in the non-TGCV group. Abbreviations: ANCOVA, analysis of covariance; ISLL, in-stent late loss; IQR, interquartile range; TGCV, triglyceride deposit cardiomyovasculopathy.

After adjusting for sex and TGCV diagnosis using ANCOVA, in-stent late loss was significantly higher in the TGCV group than in the non-TGCV group (1.17 mm [0.30, 2.06] vs. 0.29 mm [0.21, 0.41]; *p* = 0.045). Comparing the cumulative distribution curves between the 2 groups showed that the distribution of in-stent late loss was greater in the TGCV group than in the non-TGCV group (Figure 2).

### In-stent restenosis and target lesion revascularization

**Figure 3.**
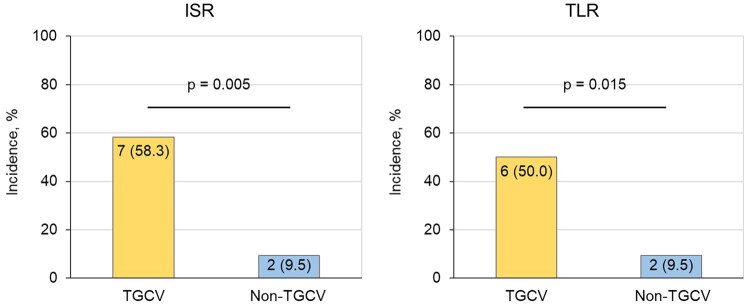
Restenosis and revascularization data. Figure (a) on the left shows the incidence of ISR. Figure (b) on the right shows the incidence of TLR. The P-values were calculated using the Fisher’s exact test. Abbreviations: ISR, in-stent restenosis; TGCV, triglyceride deposit cardiomyovasculopathy; TLR, target lesion revascularization.

ISR and TLR rates were significantly higher in the TGCV group than in the non-TGCV group (58.3% vs. 9.5%; *p* = 0.005 and 50.0% vs. 9.5%; *p* = 0.015, respectively) (Figure 3). TLR developed in 6 out of 12 lesions in the TGCV group and in 2 out of 21 lesions in the non-TGCV group (50.0% vs. 9.5%; *p* = 0.015). Univariate analysis revealed that hypertension and TGCV were predictive factors for ISR and TLR (Table 3). Multivariate analysis after adjusting for the confounding factors for ISR and TLR showed that TGCV was an independent predictive factor for ISR and TLR (Table 4). For reference, exploratory subgroup cross-tabulations stratified by DES type and lesion complexity are presented in Supplemental Table S2; these analyses are descriptive only due to the small cell counts.

**Table 3.** Results of univariate logistic regression analysis for identification of predictive risk factors for restenosis and revascularisation.

Variables	ISR		TLR	
	OR (95% CI)	p value	OR (95% CI)	p value
Male sex	0.29 (0.05–1.80)	0.182	0.57 (0.08–3.92)	0.569
Age, year	1.04 (0.96–1.13)	0.351	1.06 (0.97–1.16)	0.224
BMI, kg/m^2^	0.96 (0.83–1.10)	0.543	0.95 (0.82–1.11)	0.528
Prior MI, yes	0.88 (0.08–9.70)	0.913	1.05 (0.09–11.9)	0.970
Prior CABG*, yes	–	–	–	–
Hemodialysis period, year	1.06 (0.95–1.19)	0.314	1.05 (0.94–1.18)	0.364
Hypertension, yes	0.11 (0.02–0.80)	0.029	0.09 (0.01–0.64)	0.017
Diabetes Mellitus, yes	0.40 (0.08–1.91)	0.251	0.28 (0.05–1.49)	0.135
Dyslipidaemia, yes	1.69 (0.34–8.40)	0.520	1.31 (0.26–6.72)	0.747
Current smoker, yes	1.25 (0.27–5.83)	0.776	0.92 (0.19–4.54)	0.922
Statin use, yes	2.00 (0.40–9.91)	0.396	1.54 (0.30–7.87)	0.605
Insulin use, yes	0.86 (0.14–5.31)	0.868	0.37 (0.04–3.56)	0.387
DAPT use*, yes	–	–	–	–
P2Y12 inhibitor use, yes	0.15 (0.01–1.94)	0.147	0.13 (0.01–1.62)	0.112
BMIPP wash-out rate, %	0.97 (0.93–1.01)	0.086	0.98 (0.95–1.02)	0.424
Diffuse narrowing coronary artery*, yes	–	–	–	–
LVEF, %	1.01 (0.95–1.08)	0.683	1.00 (0.94–1.07)	0.978
TGCV, yes	13.3 (2.08–58.0)	0.006	9.50 (1.50–60.1)	0.017
AHA/ACC type B2/C*, yes	–	–	–	–
Bifurcation lesion, yes	0.59 (0.12–2.93)	0.520	0.76 (0.15–3.92)	0.747
DES type*	–	–	–	–
Stent length, mm	0.91(0.78–1.06)	0.210	0.91 (0.77–1.06)	0.223
Stent diameter, mm	1.71 (0.22–13.0)	0.606	3.57 (0.40–31.8)	0.254
Lesion length, mm	0.94(0.80–1.11)	0.487	0.94 (0.79–1.12)	0.506
Reference diameter, mm	1.01 (0.22–4.66)	0.988	2.06 (0.42–10.2)	0.375
Pre MLD, mm	0.34 (0.05–2.19)	0.255	0.46 (0.07–3.02)	0.422
Pre %DS, %	1.04 (0.98–1.10)	0.195	1.05 (0.99–1.12)	0.133
Post MLD, mm	1.21 (0.31–4.83)	0.784	2.14 (0.50–9.12)	0.304
Post %DS, %	0.98 (0.86–1.11)	0.707	0.95 (0.83–1.09)	0.467
Duration to F/U CAG, month	0.99 (0.82–1.19)	0.888	1.06 (0.83–1.22)	0.949

* Logistic regression analyses for prior CABG, DAPT use, diffuse narrowing coronary artery, AHA/ACC type B2/C, and DES type were not conducted (–) because ISR and TLR did not occur in patients without prior CABG, in those not presenting diffuse narrowing coronary artery and AHA/ACC type B2/C, in those implanted with Sirolimus and Everolimus-eluting stents, and in those not receiving DAPT.

Abbreviations: BMI, body mass index; CI, confidence interval; DES, drug-eluting stent; DS, diameter stenosis; F/U, follow-up; ISR, in-stent restenosis; MI, myocardial infarction; MLD, minimal lumen diameter; TGCV, triglyceride deposit cardiomyovasculopathy; TLR, target lesion revascularization.

**Table 4.** Results of multivariate logistic regression analysis for identification of predictive risk factors for restenosis and revascularisation.

ISR		
Variable	OR (95% CI)	p-value
Model 1		
TGCV, yes	15.4 (1.64–144)	0.017
Age, year	0.98 (0.89–1.09)	0.750
Male sex, yes	0.99 (0.11–9.27)	0.999
Model 2		
TGCV, yes	11.0 (1.47–81.7)	0.020
Hypertension, yes	0.16 (0.02–1.63)	0.112
Diabetes Mellitus, yes	1.02 (0.14–7.63)	0.985
Model 3		
TGCV, yes	18.1 (2.23–146)	0.007
Insulin use, yes	1.73 (0.16–18.8)	0.652
HD periods, year	1.10 (0.96–1.24)	0.163
Model 4		
TGCV, yes	15.0 (2.11–107)	0.007
Stent diameter, mm	0.89 (0.73–1.07)	0.216
Reference diameter, mm	0.89 (0.11–7.23)	0.911
	TLR	
Variable	OR (95% CI)	p-value
Model 1		
TGCV, yes	11.2 (1.28–98.4)	0.029
Age, year	1.02 (0.91–1.13)	0.760
Male sex, yes	2.17 (0.23–20.9)	0.504
Model 2		
TGCV, yes	6.62 (0.87–50.2)	0.067
Hypertension, yes	0.13 (0.01–1.25)	0.078
Diabetes Mellitus, yes	0.60 (0.08–4.44)	0.614
Model 3		
TGCV, yes	10.8 (1.48–78.9)	0.019
Insulin use, yes	0.49 (0.04–6.13)	0.576
HD periods, year	1.07 (0.95–1.22)	0.263
Model 4		
TGCV, yes	12.3 (1.62–93.2)	0.015
Stent diameter, mm	0.92 (0.77–1.10)	0.345
Reference diameter, mm	2.67 (0.35–20.1)	0.342

Abbreviations: CI, confidence interval HD, hemodialysis; ISR, in-stent late loss; OR, odds ratio; TGCV, triglyceride deposit cardiomyovasculopathy; TLR, target lesion revascularisation.

### Other cardiovascular events

The ratios of cardiovascular events were not significantly different between the TGCV and non-TGCV groups (2 out of 9, 22.2% vs. 2 out of 17, 11.8%; *p* = 0.591). The details of the cardiovascular events in the TGCV and non-TGCV groups were as follows: acute myocardial infarction (1 out of 9, 11.1% vs. 1 out of 17, 5.9%; *p* > 0.999), heart failure (1 out of 9, 11.1% vs. 1 out of 17, 5.9%; *p* > 0.999), cerebral hemorrhage (0.0% vs. 0.0%), and sudden death (0.0% vs. 0.0%).

### Representative case

**Figure 4.**
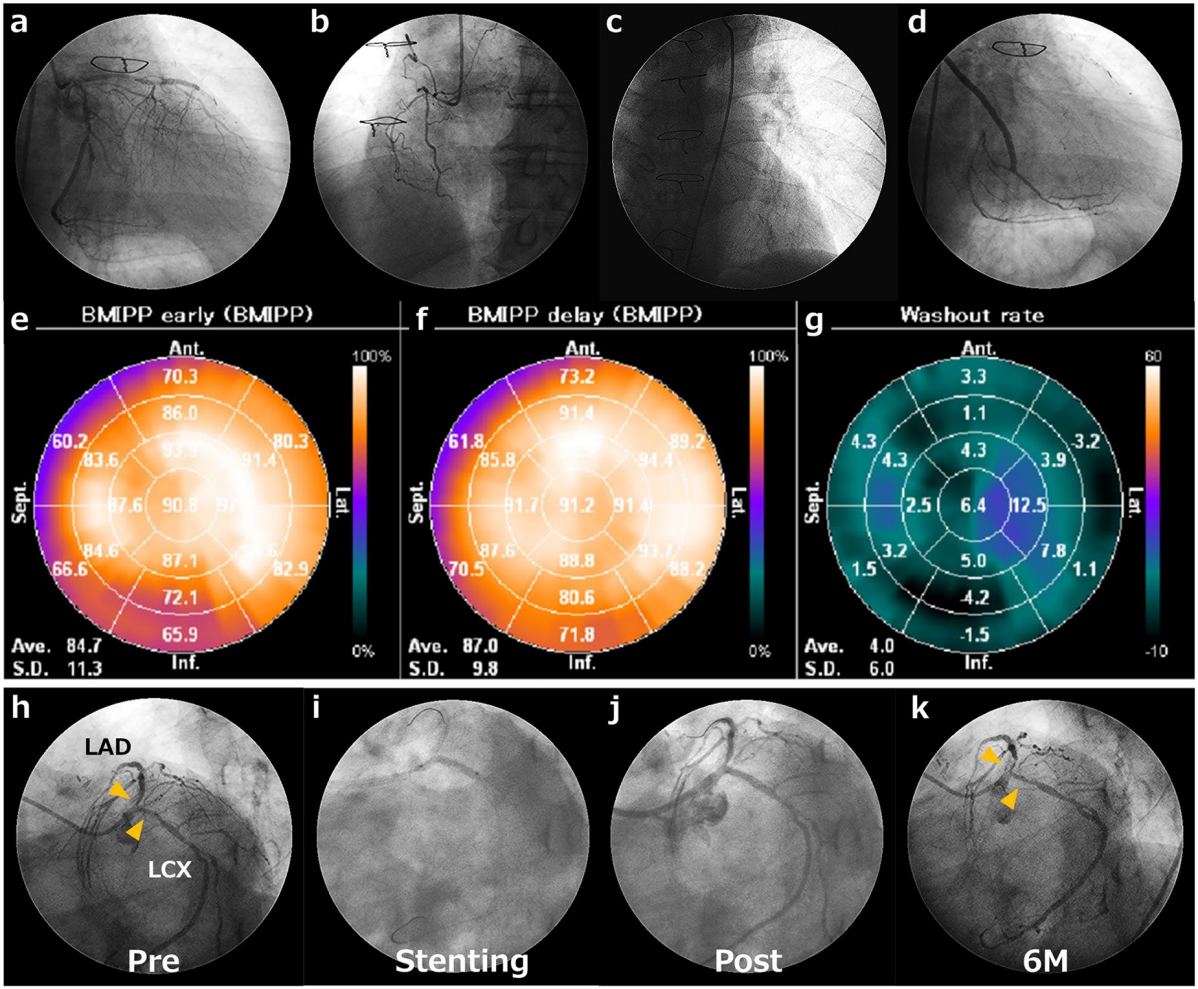
Representative case. Figure (a–d) show diffuse narrowing and occlusive left coronary artery, occlusive right coronary artery, left internal thoracic artery graft connected to LAD, and saphenous vein graft connected to the right coronary artery, respectively. Figure (e–g) shows the early phase, late phase, and washout rate of BMIPP, respectively. The washout rate is decreased and shows 4.0%. Stents implanted at the proximal of the LAD and LCX (h–j) resulted in restenosis six months later (k). Abbreviations: LAD, left anterior descending artery; LCX, left circumflex artery

Figure 4 shows a case of a 75-year-old man on HD with a history of coronary artery bypass grafting. This case was diagnosed with TGCV by the following items based on diagnostic criteria for TGCV (Supplementary Table S1) [10,14]: Diffuse narrowing of coronary arteries documented by CAG as a major item and decreased washout rate (<10%) in myocardial 123I-BMIPP SPECT as an essential item. Coronary stents were implanted for the stenosis in the proximal left anterior descending and left circumflex arteries with angina; however, six months later, he developed chest pain, and stent restenosis was confirmed.

## Discussion

### Main findings

In the current study, a smaller MLD and higher in-stent late loss at the 1-year follow-up, with a subsequent increase in the incidences of ISR (58.3%) and TLR (50.0%), were observed in the TGCV group compared to the non-TGCV group. Furthermore, TGCV was found to be an independent predictor of ISR and TLR, even after adjusting for confounding factors.

### Interpretation of findings

In this preliminary study, TGCV was associated with significantly greater in-stent late loss and higher rates of ISR and TLR after DES implantation in patients on HD. These findings suggest that TGCV may represent a distinct substrate for DES failure in this high-risk population. Given the unique metabolic and vascular features of TGCV—including defective intracellular TG hydrolysis and diffuse concentric coronary narrowing—TGCV may predispose to exaggerated neointimal proliferation and impaired vascular healing after PCI.

While our results support a potential mechanistic link between TGCV and restenosis, they should be interpreted as hypothesis-generating rather than definitive. This is particularly important considering the small sample size and the limited number of outcome events, which restrict statistical power and precision. Nevertheless, the observed consistency across angiographic and clinical endpoints highlights the clinical relevance of TGCV in HD patients undergoing PCI and underscores the need for improved diagnostic recognition and tailored therapeutic approaches.

### Effects of drug-eluting stents on triglyceride deposit cardiomyovasculopathy

CAD in TGCV reportedly exhibits unique coronary artery findings with TG deposits in smooth muscle cells, characterized by diffuse, narrowing, concentric-type stenosis (Figure 4) [8,15,16]. DESs have become the most common treatment strategy, even for treating complex lesions. Put simply, DESs are superior to bare metal stents in reducing in-stent late loss of complex lesions, such as diffuse, longer, and calcified target lesions [17,18]. Nevertheless, in the present study, in-stent late loss at the 1-year follow-up showed a four-fold increase in the TGCV group (1.17 mm) than the non-TGCV group (0.29 mm). In-stent late loss previously reported among HD patients without TGCV was 0.27 mm [19,20], consistent with the present study’s results. By contrast, TGCV demonstrated substantially greater late loss, underscoring the potential clinical relevance of this signal despite statistical imprecision. These results suggest that TGCV limits the efficacy of DESs exclusively in HD patients with CAD.

### Plausible mechanisms for the clinical deficiency of drug-eluting stents in triglyceride deposit cardiomyovasculopathy

ISR comes from not only the inflammatory response of the vessels but also from the recovery process [21]. Macrophages activated by endothelial damage due to stent implantation are the primary source of proinflammatory cytokines and growth factors that stimulate systemic inflammation [22]. Additionally, several factors associated with endothelial function, collagen synthesis, and metabolism are involved in ISR; this is even more so in HD cases [23–25]. Conversely, evidence suggests that the factors relevant to ISR, including vascular endothelial growth factor-alpha, tumor necrosis factor-alpha, transforming growth factor-beta, interleukin-6, lipoprotein lipase, and glucokinase, exhibit distinct expression levels in mouse models of TGCV than those in wild-type mice [26,27]. These findings may partially explain the clinical deficiency of DESs in patients with TGCV.

There also remains the possibility that the mammalian target of rapamycin (mTOR) inhibitors in DESs might locally affect TG metabolism in TG-harboring smooth muscle cells and other vascular cells within the target lesion in TGCV [12]. It was reported that, in TGCV patients with deficient intracellular TG hydrolysis, lipophagy, an autophagic process that targets and degrades lipid droplets to rescue cellular energy failure, was upregulated in skeletal muscle specimens [28,29]. Given that autophagy is regulated by mTOR signaling [30], mTOR inhibition with DESs might affect this type of compensatory lipid degradation and promote TG deposition in coronary arteries in TGCV, although the *de novo* expression and regulation of mTOR signaling in vascular cells remain unknown. These findings suggest that a different mechanism of ISR exists in the TGCV group compared to the non-TGCV group.

In summary, preclinical studies also indicate that TG accumulation may modify smooth muscle cell phenotype and inflammatory signaling, potentially facilitating restenosis [26,27]. Furthermore, mTOR inhibitors used in DES may interfere with compensatory lipophagy-mediated lipid degradation in TGCV [28–30], potentially enhancing TG deposition within the coronary arterial wall. These mechanisms may partially explain the higher ISR incidence observed in TGCV; however, they remain hypothesis-generating and require mechanistic validation. We present this as a hypothesis for future experimental testing.

### Paradigm shift in the disease concept of hemodialysis-related coronary artery disease

Currently available medical and therapeutic treatments, such as statins, PCI, and coronary artery bypass graft, have little effect on HD-related CAD and improvement of prognosis. Furthermore, we recently reported the relatively high comorbidity of TGCV in HD patients and the relationship between a novel type of podocytopathy and proteinuria in TGCV patients [11,31]. Combined with these findings, the assessment of TGCV in HD patients could allow us to better understand the appropriate treatment for this population. In addition, given its specific effect on atherosclerosis, TGCV poses a significant therapeutic challenge when treating PCI, exclusively among HD patients. After obtaining a pre-clinical proof of concept using a mouse model for TGCV [32], the supplemental intake of tricaprin, a class of medium-chain TGs, has been shown to facilitate LCTG lipolysis, relieve symptoms, and improve the long-term survival rate [33]. Future studies are required to identify the potential benefits of tricaprin when treating ISR in patients with TGCV.

## Limitations

This study has several limitations. The most important limitation is the small sample size with few outcome events, which yielded wide CIs and imprecision in effect estimates. Accordingly, all conclusions are preliminary and hypothesis-generating. Second, this single-center study in Japan limits generalizability, particularly because diagnosis of TGCV relied on 123I-BMIPP scintigraphy—a modality not widely available outside Japan—raising concerns about selection bias [34]. Therefore, screening methods are needed to identify patients with suspected TGCV. Moreover, many screened patients were excluded for not undergoing PCI or follow-up angiography, potentially enriching for patients with angiographically confirmed disease or surveillance-driven outcomes and thereby inflating or deflating effect sizes. Medication exposure beyond antiplatelets and statins (e.g., chronic anti-inflammatory agents) was incompletely captured and not controlled, which could confound ISR/TLR risk. We lacked uniform laboratory data on mineral metabolism (calcium, phosphate, PTH), precluding adjustment for these HD-specific determinants of restenosis. Given the limited number of outcome events, we emphasized consistency between univariate estimates and multivariable models; however, the possibility of model overfitting cannot be excluded. In-stent late loss was not evaluated using an intravascular imaging modality; therefore, it remains unclear whether in-stent late loss was caused by neointimal proliferation, neoatherosclerosis, thrombus, or stent underexpansion. This is a particularly important point given the pathophysiology proposed for TGCV, and the absence of such data represents a significant limitation. Future studies incorporating OCT/IVUS and, where feasible, histopathology are needed to validate mechanistic hypotheses. Finally, to improve external validity, future studies should adopt multicenter designs and consider an appropriate case-control sampling strategy, or alternatively, prospective multicenter registries with standardized data collection.

## Conclusions

Our findings suggest an association between TGCV and vascular failure after DES implantation in patients on HD. Although TGCV may represent a potential therapeutic target in this population, these preliminary observations require confirmation in prospective studies with larger patient cohorts.

## Supplementary Material

→→TGCV透析論文_Manuscript_Suppl●_revise01 (1).docx

## Data Availability

The datasets used and/or analyzed during the current study are available from the corresponding author on reasonable request.
