# Supplementary material for: Triglyceride deposit cardiomyovasculopathy as a predictor of vascular failure after coronary intervention in patients with hemodialysis: a preliminary analysis
Source: Ren Fail. 2026 Apr 22;48(1):2650580. doi: 10.1080/0886022X.2026.2650580 (PMC13103981; doi:10.1080/0886022X.2026.2650580)
Supplement: →→TGCV透析論文_Manuscript_Suppl●_revise01 (1).docx [file IRNF_A_2650580_SM0049.docx]

**Supplemental Material**

**Contents**

Supplemental Table S1. Diagnostic Criteria 2020 for TGCV

Supplemental Table S2. Subgroup Cross-tabulations Stratified by DES Types and Lesion Complexity

Supplementary Text S1. [123I]-β-methyl iodophenyl-pentadecanoic acid scintigraphy

References

**Supplemental Table S1.**

**Diagnostic Criteria 2020 for TGCV** ^1^

| Items | Clinical findings |
| --- | --- |
| 1. Essential items | Impaired LCFA metabolism or TG deposition in myocardium |
|  | 1) Decreased washout rate (<10%) in myocardial ^123^I- BMIPP SPECT |
|  | 2) Myocardial TG deposition by biopsy specimens (a) |
|  | 3) Myocardial TG deposition by CT or MR spectroscopy |
| 2. Major items | 1) Decreased left ventricular ejection fraction (<40%) |
|  | 2) Diffuse narrowing of coronary arteries documented by CAG and/or coronary CT angiography (b) |
|  | 3) Typical Jordans' anomaly (apparent vacuoles of about 1 μm in size) of polymorphonuclear leucocytes in peripheral blood smear (c) ^2^ |

Diagnosis

Definite TGCV: One or more essential items and one or more major items are met.

Probable TGCV: At least one essential item is met.

Supportive items (d)

1) Diabetes mellitus (e)

2) Hemodialysis

(a) For tissue TG contents examination, frozen sections with osmium fixation but no paraffin sections, should be used for prevention of lipid elution

(b) The presence or absence of a significant stenosis is not considered

(c) For difficult cases, May-Giemsa staining slides of peripheral blood smear will be evaluated by the Japan TGCV study group

(d) These items are commonly present according to autopsy heart analysis and small-scale cohort analysis performed by the Japan TGCV study group, but are not proven to have diagnostic accuracy or a causal relationship.

(e) According to the diagnostic criteria of diabetes mellitus by the Japan Diabetes Society ^3^

Abbreviations: CAG: coronary angiography; CT: computed tomography; LCFA: long chain fatty acid; MR: magnetic resonance; SPECT: single-photon emission computed tomography; TG: triglyceride; TGCV: Triglyceride deposit cardiomyovasculopathy; ^123^I-BMIPP: iodine-123-β-methyl iodophenyl-pentadecanoic acid.

**Supplemental Table S2.**

**Subgroup Cross-tabulations Stratified by DES Types and Lesion Complexity**

| No | Group | Late loss, mm | ISR | TLR | TypeB2/C | DES types |
| --- | --- | --- | --- | --- | --- | --- |
| 1 | TGCV | 2.79 | yes | yes | yes | Everolimus-eluting stent |
| 2 | TGCV | 2.24 | yes | yes | yes | Everolimus-eluting stent |
| 3 | TGCV | 2.13 | yes | yes | yes | Everolimus-eluting stent |
| 4 | TGCV | 2.04 | yes | yes | yes | Everolimus-eluting stent |
| 5 | TGCV | 1.73 | yes | yes | yes | Everolimus-eluting stent |
| 6 | TGCV | 1.56 | yes | yes | yes | Everolimus-eluting stent |
| 7 | TGCV | 0.78 | yes | no | yes | Sirolimus-eluting stent |
| 8 | TGCV | 0.48 | no | no | yes | Everolimus-eluting stent |
| 9 | TGCV | 0.35 | no | no | yes | Everolimus-eluting stent |
| 10 | TGCV | 0.15 | no | no | yes | Everolimus-eluting stent |
| 11 | TGCV | 0.07 | no | no | yes | Everolimus-eluting stent |
| 12 | TGCV | 0.04 | no | no | yes | Zotarolimus-eluting stent |
| 1 | non-TGCV | 2.97 | yes | yes | yes | Everolimus-eluting stent |
| 2 | non-TGCV | 2.02 | yes | yes | yes | Everolimus-eluting stent |
| 3 | non-TGCV | 0.6 | no | no | yes | Zotarolimus-eluting stent |
| 4 | non-TGCV | 0.54 | no | no | yes | Everolimus-eluting stent |
| 5 | non-TGCV | 0.45 | no | no | yes | Zotarolimus-eluting stent |
| 6 | non-TGCV | 0.41 | no | no | yes | Everolimus-eluting stent |
| 7 | non-TGCV | 0.4 | no | no | yes | Everolimus-eluting stent |
| 8 | non-TGCV | 0.38 | no | no | yes | Everolimus-eluting stent |
| 9 | non-TGCV | 0.35 | no | no | yes | Everolimus-eluting stent |
| 10 | non-TGCV | 0.33 | no | no | yes | Everolimus-eluting stent |
| 11 | non-TGCV | 0.29 | no | no | yes | Biolimus-eluting stent |
| 12 | non-TGCV | 0.29 | no | no | yes | Everolimus-eluting stent |
| 13 | non-TGCV | 0.28 | no | no | yes | Biolimus-eluting stent |
| 14 | non-TGCV | 0.25 | no | no | no | Everolimus-eluting stent |
| 15 | non-TGCV | 0.24 | no | no | yes | Everolimus-eluting stent |
| 16 | non-TGCV | 0.21 | no | no | no | Biolimus-eluting stent |
| 17 | non-TGCV | 0.16 | no | no | yes | Everolimus-eluting stent |
| 18 | non-TGCV | 0.1 | no | no | yes | Everolimus-eluting stent |
| 19 | non-TGCV | 0.1 | no | no | yes | Zotarolimus-eluting stent |
| 20 | non-TGCV | 0.09 | no | no | yes | Paclitaxel-eluting stent |
| 21 | non-TGCV | 0.05 | no | no | no | Zotarolimus-eluting stent |

TGCV, triglyceride deposit cardiomyovasculopathy; ISR, in-stent late loss; TLR, target lesion revascularisation; DES, drug-eluting stent

**Supplemental Text S1.**

**[123I]-β-methyl iodophenyl-pentadecanoic acid scintigraphy**

[123I]-β-methyl iodophenyl-pentadecanoic acid (BMIPP) is useful for single-photon emission computed tomography (SPECT) imaging to evaluate long-chain fatty acid (LCFA) metabolism because of its high uptake through CD36 and long retention in the myocardium. LCFAs are an essential energy source in the normal heart, and are taken up through CD36 and transported to mitochondria for β-oxidation to produce ATP. BMIPP is an LCFA^4^ with similar intracellular dynamics to other LCFAs.^5^ In normal subjects, after their uptake by cardiomyocytes, LCFAs are either used to synthesize triglycerides, or undergo β-oxidation to be utilized for energy production. However, in patients with triglyceride deposit cardiomyovasculopathy (TGCV), because of insufficient hydrolysis of intracellular triglycerides to supply LCFAs, once LCFAs are pooled as triglycerides, they remain inside the cell without hydrolysis.^6^ Therefore, patients with TGCV have a markedly decreased washout rate (WOR) of BMIPP,^7^ reflecting a defective myocardial LCFA and triglyceride metabolism. To calculate the WOR of BMIPP, acquisition of delayed imaging up to 240 min after the injection of a tracer is performed, in addition to early imaging at <30 min. WOR is calculated from the mean tracer counts after constructing polar map displays from short-axis, early, and delayed SPECT imagings.^7,8^ The WOR of BMIPP provides information on the myocardial metabolism of LCFAs; therefore, BMIPP scintigraphy is an important nuclear imaging method for the diagnosis of TGCV.

**References**

1. K. Kobayashi, Y. Sakata, H. Miyauchi, Ikeda Y, Nagasawa Y, Nakajima K, Shimada K, Kozawa J, Hao H, Amano T, Yoshida H, Inaba T, Hashimoto C, Hirano K. The diagnostic criteria 2020 for triglyceride deposit Cardiomyovasculopathy. *Annals of Nuclear Cardiology* 2020;6:99–104. https://doi.org/10.17996/anc.20-00131.

2. Jordans GH. The familial occurrence of fat containing vacuoles in the leukocytes diagnosed in two brothers suffering from dystrophia musculorum progressiva (ERB.). *Acta Med Scand* 1953;145(6):419-423.

3. Committee of the Japan Diabetes Society on the Diagnostic Criteria of Diabetes Mellitus, Seino Y, Nanjo K, Tajima N, Kadowaki T, Kashiwagi A, Araki E, Ito C, Inagaki N, Iwamoto Y, Kasuga M, Hanafusa T, Haneda M, Ueki K. Report of the Committee on the classification and diagnostic criteria of diabetes mellitus. *Diabetology International* 2010;1(1):2-20.

4. Goodman MM, Kirsch G, Knapp FF, Jr. Synthesis and evaluation of radioiodinated terminal p-iodophenyl-substituted alpha- and beta-methyl-branched fatty acids*. J Med Chem* 1984;27(3):390-397.

5. Knapp FF, Jr., Ambrose KR, Goodman MM. New radioiodinated methyl-branched fatty acids for cardiac studies. *Eur J Nucl Med* 1986;12 Suppl:S39-44.

6. Hirano K, Tanaka T, Ikeda Y, Yamaguchi S, Zaima N, Kobayashi K, Suzuki A, Sakata Y, Sakata Y, Kobayashi K, Toda T, Fukushima N, Ishibashi-Ueda H, Tavian D, Nagasaka H, Hui SP, Chiba H, Sawa Y, Hori M. Genetic mutations in adipose triglyceride lipase and myocardial up-regulation of peroxisome proliferated activated receptor-gamma in patients with triglyceride deposit cardiomyovasculopathy. *Biochem Biophys Res Commun* 2014;443(2):574-579.

7. Miyauchi H, Hashimoto C, Ikeda Y, Li M, Nakano Y, Kozawa J, Sai E, Nagasawa Y, Sugimura K, Kinugawa S, Kawaguch K, Shimada K, Ide T, Amano T, Higashi M, Inaba T, Nakamura H, Kobayashi K, Hirano K. Diagnostic criteria and severity score for triglyceride deposit cardiomyovasculopathy. *Annals of Nuclear Cardiology* 2018;4(1):94-100.

8. Hirano K, Ikeda Y, Sugimura K, Sakata Y. Cardiomyocyte steatosis and defective washout of iodine-123-beta-methyl iodophenyl-pentadecanoic acid in genetic deficiency of adipose triglyceride lipase. *Eur Heart J* 2015;36(9):580.
